# Elevated dietary zinc oxide levels do not have a substantial effect on porcine reproductive and respiratory syndrome virus (PPRSV) vaccination and infection

**DOI:** 10.1186/1743-422X-11-140

**Published:** 2014-08-08

**Authors:** Weidong Chai, Zhenya Wang, Pawel Janczyk, Sven Twardziok, Ulrike Blohm, Nikolaus Osterrieder, Michael Burwinkel

**Affiliations:** Institut für Virologie, Freie Universität Berlin, Robert-von-Ostertag-Str. 7-13, 14163 Berlin, Germany; Bundesinstitut für Risikobewertung, Abteilung für Biologische Sicherheit, Fachgruppe für Molekulare Diagnostik und Genetik, Diedersdorfer Weg 1, 12277 Berlin, Germany; Molekularbiologie und Bioinformatik, Charité - Universitätsmedizin Berlin, Arnimallee 22, 14195 Berlin, Germany; Institut für Immunologie, Friedrich-Loeffler-Institut, Südufer 10, 17493 Greifswald-Insel Riems, Germany

**Keywords:** PRRSV, Inactivated vaccine, Dietary zinc oxide

## Abstract

**Background:**

Porcine reproductive and respiratory syndrome virus (PRRSV) is one of the most important infectious agents for the swine industry worldwide. Zinc (Zn) salts, which are widely used as a dietary supplement in swine nutrition, have shown antiviral effects in vitro as well as in vivo. The purpose of this study was to determine the influence of dietary zinc oxide supplementation on vaccination and challenge infection with PRRSV.

**Findings:**

The clinical course of PRRS and the success of vaccination with an experimental inactivated vaccine were compared between animals receiving a conventional diet (50 ppm Zn, control group) and diets supplemented with Zn oxide (ZnO) at final Zn concentrations of 150 or 2,500 ppm. Pigs receiving higher dietary Zn levels showed a tendency towards higher neutralizing antibody levels after infection, while dietary Zn levels did not substantially influence the number of antiviral IFN-gamma secreting cells (IFN-gamma-SC) or percentages of blood immune cell subsets after infection. Finally, feeding higher dietary Zn levels reduced neither clinical symptoms nor viral loads.

**Conclusions:**

Our results suggest that higher levels of dietary ZnO do not have the potential to stimulate or modulate systemic immune responses after vaccination and heterologous PRRSV infection to an extent that could improve the clinical and virological outcome.

**Electronic supplementary material:**

The online version of this article (doi:10.1186/1743-422X-11-140) contains supplementary material, which is available to authorized users.

## Findings

### Introduction

Porcine reproductive and respiratory syndrome (PRRS) is one of the most significant swine diseases worldwide [1]. Efficient PRRS virus (PRRSV) vaccines would be invaluable in minimizing the clinical and economic impact of PRRSV infections, but currently safe and effective vaccines which protect against a wide variety of strains are not available [2].

Zinc (Zn) ion salts exhibit a broad-spectrum antiviral activity against a variety of viruses in vitro, including the animal viruses equine arteritis virus and transmissible gastroenteritis virus [3, 4]. In the European Union standard dietary Zn levels in feedingstuffs are limited to 150 ppm due to environmental reasons. However, in other countries high levels of in-feed Zn oxide (ZnO, 2,000-3,000 ppm) may be added to the diet of pigs during a restricted period following weaning to prevent post-weaning diarrhea [5] as high levels of ZnO have been proven to conserve the intestinal flora during the critical period following the change of diet that place at weaning [6]. Despite this effect, the exact mechanisms of ZnO action remain uncertain, and the local or systemic effects of ZnO against specific viral pathogens also remain largely unknown.

We evaluated the systemic effects of different Zn levels added to a conventional diet containing 50 ppm Zn (Zn^low^, control group) against PRRSV. Two other groups were fed the diet supplemented with ZnO at final concentrations of 150 ppm Zn (Zn^med^), or 2,500 ppm Zn (Zn^high^). Half of the animals received a single vaccination with an experimental UV-inactivated type I PRRSV (Lelystad virus; LV), since it was shown that a similar vaccination with such a virus in combination with a suitable adjuvant could strongly prime the virus-neutralizing (VN) response and reduce duration of viremia after homologous challenge [7]. In contrast to Vanhee et al., we chose a single-vaccination approach and challenge-infected the animals with a heterologous type I PRRSV (95,38% sequence identity for the envelope glycoprotein GP5, which bears a major neutralizing epitope) in order test the influence of elevated Zn levels on an suboptimal antigen stimulus and on cross-protection.

## Methods

The study was approved by the local animal welfare authority (Landesamt für Gesundheit und Soziales, Berlin, Germany) under the registration number G 0116/12. German Landrace piglets (n = 72) of both sexes from a PRRSV-free herd were weaned at the age of 28 days, moved to a biosafety level 2 experimental facility (Bundesinstitut für Risikobewertung, Berlin, Germany), and randomly allocated to six pens (n = 12 per pen). Piglets were assigned to three different diets (2 pens per diet). At the age of 63 days, the animals receiving the Zn^high^ diet were switched to the Zn^med^ diet, in order to avoid toxic effects of Zn. One week after commencing the different diets, one pen per diet was chosen randomly and the animals were vaccinated intramuscularly with inactivated LV (accession M96262; kindly provided by Prof. H. Nauwynck (Ghent University, Ghent, Belgium)). Four weeks after vaccination, at the age of 63 days, all pigs were challenged with PRRSV field strain CReSA 3267 (accession JF276435; kindly provided by Prof. J. Segalés and Prof. E. Mateu (CReSA, Barcelona, Spain). Animals were infected by intranasal application of 1 ml of virus suspension with a titer of 5 × 10^6^ TCID_50_/ml to each nostril using a spray nebulizer.

Pigs were monitored daily for the presence of clinical signs and body weights were recorded weekly. Blood samples were collected weekly after vaccination and at 0, 4, 7, 14, 21, 28, and 35 days post infection (dpi). Nasal swabs were taken on the same dpi as blood samples for quantification of virus shedding. All pigs were necropsied on day 35 pi. Lung and lymphoid tissues were evaluated by visual inspection for macroscopic lesions, and samples from lungs, lymph nodes, tonsils, and spleen were taken and immediately frozen in liquid nitrogen and stored at −70°C.

For virological analysis, serum samples (4, 7, 14, 21, 28 dpi), nasal swabs (4, 7, 14 dpi), and tonsil, lung and tracheobronchial lymph node samples (35 dpi) were examined by qPCR to determine PRRSV copy numbers. RNA extraction was performed using a viral RNA/DNA purification kit (Stratec) applying 200 μl of serum or 10 mg of tissue each. RNA yields and quality were determined with a NanoDrop® ND-100 spectrophotometer (NanoDrop Technologies). Reverse transcription (RT) was performed using the DyNAmo™ cDNA Synthesis Kit (Thermo Fisher Scientific). Viral loads were quantified using a TaqMan fluorescent probe-based real-time qPCR assay in an iCycler iQ™5 detection system (Bio-Rad) with primers described elsewhere [8].

PRRSV-specific IgM and IgG antibodies were measured by ELISA (Ingezim PRRS DR, Ingenasa) according to the manufacturer’s instructions. VN antibodies against PRRSV were quantified by a viral neutralization test as previously described [9]. Neutralization of PRRSV strain CReSA 3267 was examined using PRRSV GP5 specific monoclonal antibody 3H4 (Ingenasa) and Alexa Fluor™ 488 conjugated anti-mouse IgG (H + L) secondary antibody (Invitrogen).

Peripheral blood mononuclear cells (PBMC) were isolated using density centrifugation through a Ficoll gradient (LSM1077, PAA Laboratories). Samples were treated with erythrocyte lysis buffer for 5 min on ice, PBMC were washed with 10 ml of PBS with 0.2% BSA and centrifuged for 15 min at 700 × g at 4°C. In all samples, PBMC viability was confirmed using standard procedures.

The cell-mediated PRRSV-specific immune response was measured by using ELISpot for the enumeration of IFN-gamma-SC in PBMC (Mabtech). In order to compare homologous and heterologous responses, PBMC were stimulated in parallel (2.5 × 10^5^ PBMC/well, 3 wells per pig and stimulus) with CReSA 3267 or LV at a multiplicity of infection of 0.1. Unstimulated and PHA-stimulated cells (10 μg/ml) were used as negative and positive controls, respectively. IFN-gamma-SC numbers were counted using an ELISpot Reader System (A.EL.VIS GmbH).

Flow cytometry analysis was performed as described before [10] using a BD FACSCanto™ flow cytometer (BD Biosciences). Data were analyzed with FlowJo™ software (TreeStar).

## Results and discussion

**Figure 1:**
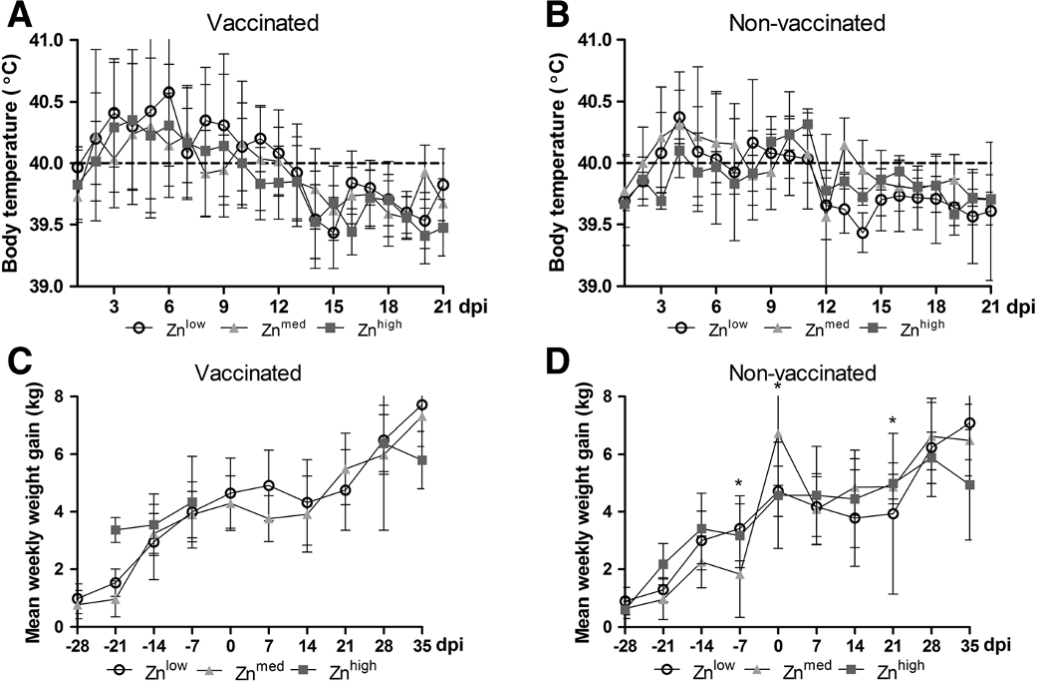
**Body temperature and weekly weight gain in pigs infected with PRRSV strain CReSA 3267. A** and **B**, Development of body temperatures. Fever (body temperatures ≥ 40°C) is indicated by the dotted line. **C** and **D** Weekly weight gains. Each bar represents the mean value ± standard deviation from 12 pigs. Asterisks indicate differences (P < 0.05) between averages at each dpi calculated by Fisher’s Least Significant Difference (*LSD*) test.

Almost all infected pigs showed clinical symptoms typical for PRRSV infection and similar to a previous study with the same PRRSV strain [11]. Increased rectal temperatures were detected for more than 14 days, and edema of the eyelids and conjunctivitis for more than 21 days. There was no significant dietary effect on fever magnitude and duration (Figure 1 A,B). Other clinical signs such as cough were observed only sporadically and lasted only for 1–2 days. Regarding the weight gain, we could only analyze the effect of the Zn^med^ diet for the vaccinated groups, given that 4 time points were missing for the pigs receiving the Zn^high^ pigs. For the remaining time points as well as for the non-vaccinated groups we found no benefits of feeding higher dietary Zn levels (Figure 1 C,D).

**Figure 2:**
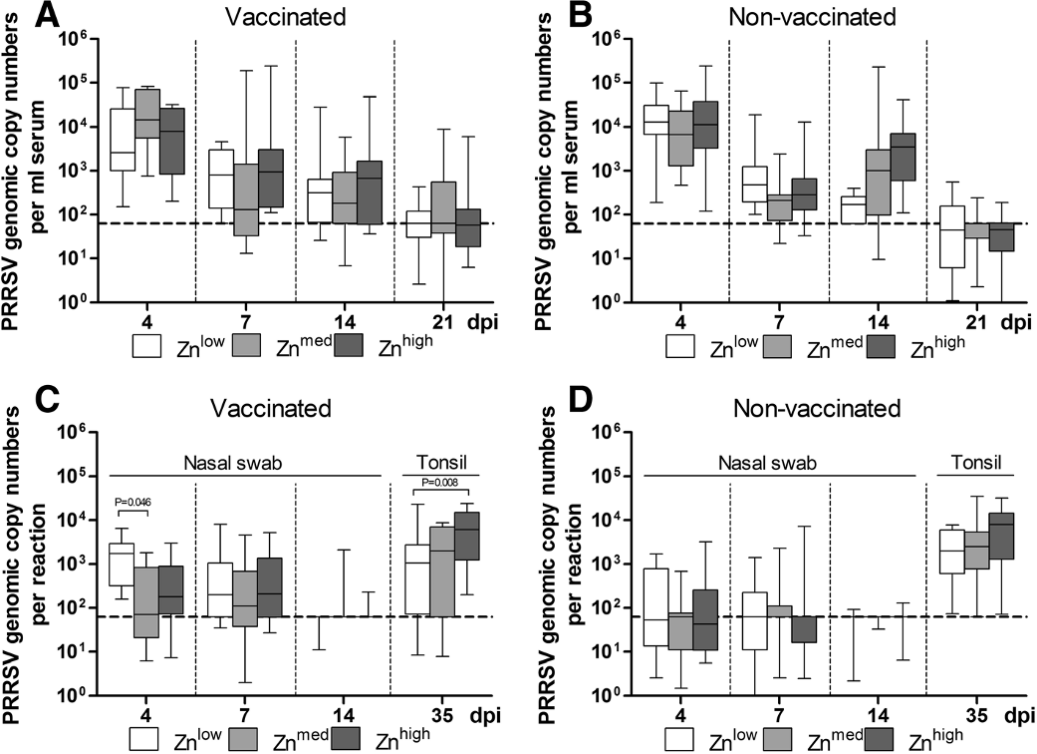
**Detection of virus in sera, nasal swabs and tonsils by quantitative RT-PCR after PRRSV infection.** Boxes indicate medians (n = 12, horizontal lines) and the lower and upper quartiles (bottoms and tops of boxes). The vertical bars in the box plots indicate the minimal and maximal values recorded. **A** and **B**, Mean viral loads (copy numbers/ml) in sera. **C** and **D**, Mean viral loads in nasal swabs and tonsils. The detection limit was 6.3 × 10^1^ copies/ml (broken line).

Mean PRRSV load in serum as determined by qPCR peaked at 4 dpi and gradually reduced later on. Neither vaccination nor elevated Zn levels showed an influence on PRRSV viremia at any time during the observation period (Figure 2 A,B). The same was observed regarding virus shedding as determined by analysis of nasal swabs (Figure 2 C,D). Persistent virus was found at 35 dpi in the majority of tonsil samples, regardless of diet, while all lung and tracheobronchial lymph node samples were tested negative for the presence of PRRSV genomes.

**Figure 3:**
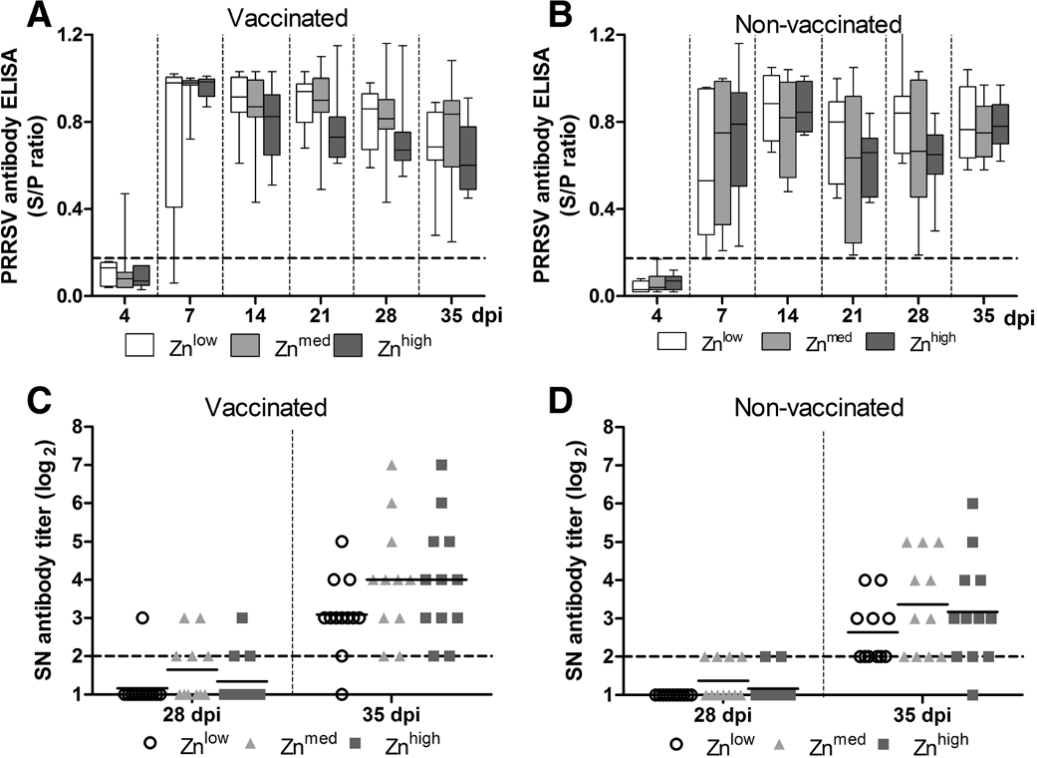
**Development of humoral responses against PRRSV. A** and **B**, PRRSV-specific antibodies measured by ELISA are shown as average sample to positive (S/P) ratios of optical density (OD) from 4 to 35 dpi. Samples with S/P ratios < 0.175 were considered negative (broken line). **C** and **D**, Serum neutralization tests were performed by a standard immunofluorescence assay and the results are shown as titers (log_2_). Samples were considered negative when the dilution was lower than 1:4 (below broken line).

PRRSV-specific IgG and IgM antibodies were not detected by ELISA in randomly chosen samples (10 vaccinated/13 non-vaccinated) before infection on day 21 before infection. On day 0, only one vaccinated animal was positive for PRRSV antibodies (Figure 3 A,B). This is in contrast with previous results showing earlier seroconversion after vaccination with UV-inactivated LV [7] and might be due to the fact that we used a cell culture-adapted non-purified LV with possibly decreased immunogenicity compared to purified LV grown on porcine alveolar macrophages used in the cited study. At 7 dpi, piglets from all groups were seropositive, except for 1 pig each in the vaccinated and non-vaccinated Zn^low^ groups. Higher (P ≤ 0.01) antibody levels were detected in vaccinated groups than in non-vaccinated groups at 7 and 21 dpi after PRRSV challenge, while the diet had no influence on antibody levels.

The generation of neutralizing antibodies is delayed in PRRSV infection and usually appears three to four weeks after infection [12]. Accordingly, in our study virus neutralizing VN antibodies were not detectable until 28 dpi in the serum. A single-vaccination with an inactivated LV did not lead to an earlier induction of VN antibodies, but vaccinated groups developed higher (P = 0.045) VN antibody titers than their non-vaccinated counterparts at 35 dpi (Figure 3C, D). A tendency towards higher VN antibody levels was evident in pigs receiving higher levels of Zn (Zn^med^) at 28 dpi. This tendency continued to 35 dpi in both Zn^med^ and Zn^high^ groups. Thus, the possibility remains that animals receiving higher dietary Zn levels might be better protected against reinfection with PRRSV.

The number of IFN-gamma-SC at 35 dpi revealed no differences after homologous or heterologous re-stimulation (Additional file 1). Flow cytometry analysis of PBMC phenotypes was performed weekly from day 0 to day 35 post infection. Single vaccination only delayed but not prevented the PRRSV-induced decrease of CD4^+^, CD8^+^ and CD4^+^CD8^+^ T cell populations, which are important for viral clearance [13]. We found no sustained effect of dietary Zn levels on any of the analysed cell subsets (Additional file 2).

Necropsies at 35 dpi revealed no gross lung lesions and lymphoid hyperplasia in tonsils, lymph nodes or spleen in any of the pigs.

Overall, the study shows that challenge infection with a wild-type PRRSV without additional environmental and social stress and the impact of secondary infections results in relatively mild clinical signs. Under these conditions, elevated dietary Zn levels could not provide enhanced protection against infection with a type I PRRSV field strain and could not improve efficacy after a single-vaccination with a heterologous inactivated vaccine.

### Availability of supporting data

The data sets supporting the results of this article are included within the article (and its additional files).

## Electronic supplementary material

Additional file 1: Figure S1: PRRSV-specific numbers of IFN-gamma-SC determined by ELISpot. PBMC collected at 35 dpi were restimulated with either of the PRRSV strains (LV or CReSA 3267) used in the study. Results are shown as average frequencies of virus-specific IFN-gamma-SC per 2.5 × 10^5^ PBMC. Filled symbols indicate results obtained after *in vitro* restimulation with the same PRRSV used for infections (homologous) while empty symbols show the results of *in vitro* restimulation with LV (heterologous). (JPEG 1 MB)

Additional file 2: Figure S2: Modulation of PBMC immune cells frequencies determined by flow cytometry analysis. A and B, Cytotoxic lymphocytes (CD3^+^CD4^−^CD8 α^high^); C and D, naïve T_H_ cells (CD3^+^CD4^+^CD8^−^); E and F, CD8^+^ γδ T cells (CD3^+^CD2^+^CD8^+^); G and H, Antibody forming and/or memory B cells (CD3^−^CD2^+^CD21^−^); I and J, NK cells. Asterisks indicate statistically significant differences (P < 0.05) between averages at each dpi. (JPEG 2 MB)
