# Supplementary figures and images for: Elevated dietary zinc oxide levels do not have a substantial effect on porcine reproductive and respiratory syndrome virus (PPRSV) vaccination and infection
Source: Virol J. 2014 Aug 8;11:140. doi: 10.1186/1743-422X-11-140 (PMC4254400; doi:10.1186/1743-422X-11-140)

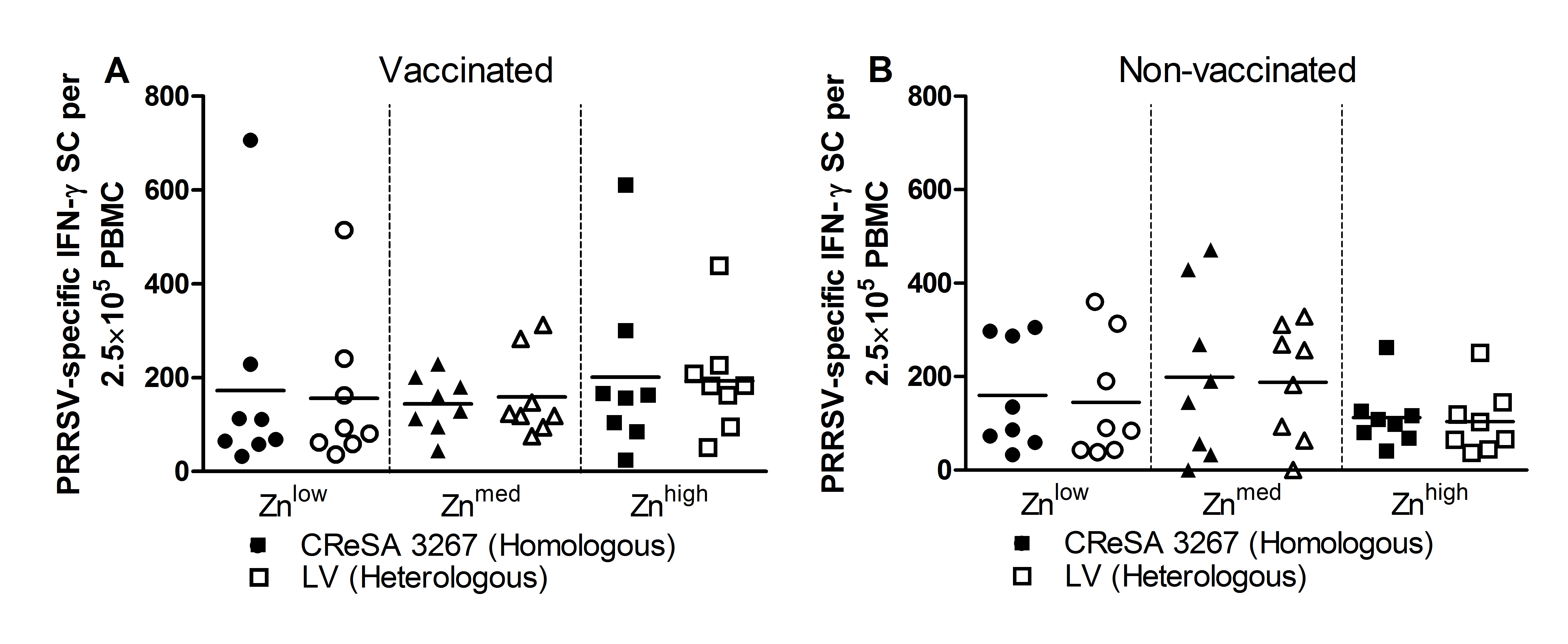

Supplement: Supplementary file 1 — Additional file 1: Figure S1: PRRSV-specific numbers of IFN-gamma-SC determined by ELISpot. PBMC collected at 35 dpi were restimulated with either of the PRRSV strains (LV or CReSA 3267) used in the study. Results are shown as average frequencies of virus-specific IFN-gamma-SC per 2.5 × 10^5^ PBMC. Filled symbols indicate results obtained after in vitro restimulation with the same PRRSV used for infections (homologous) while empty symbols show the results of in vitro restimulation with LV (heterologous). (JPEG 1 MB) [file 12985_2014_2469_MOESM1_ESM.jpeg]

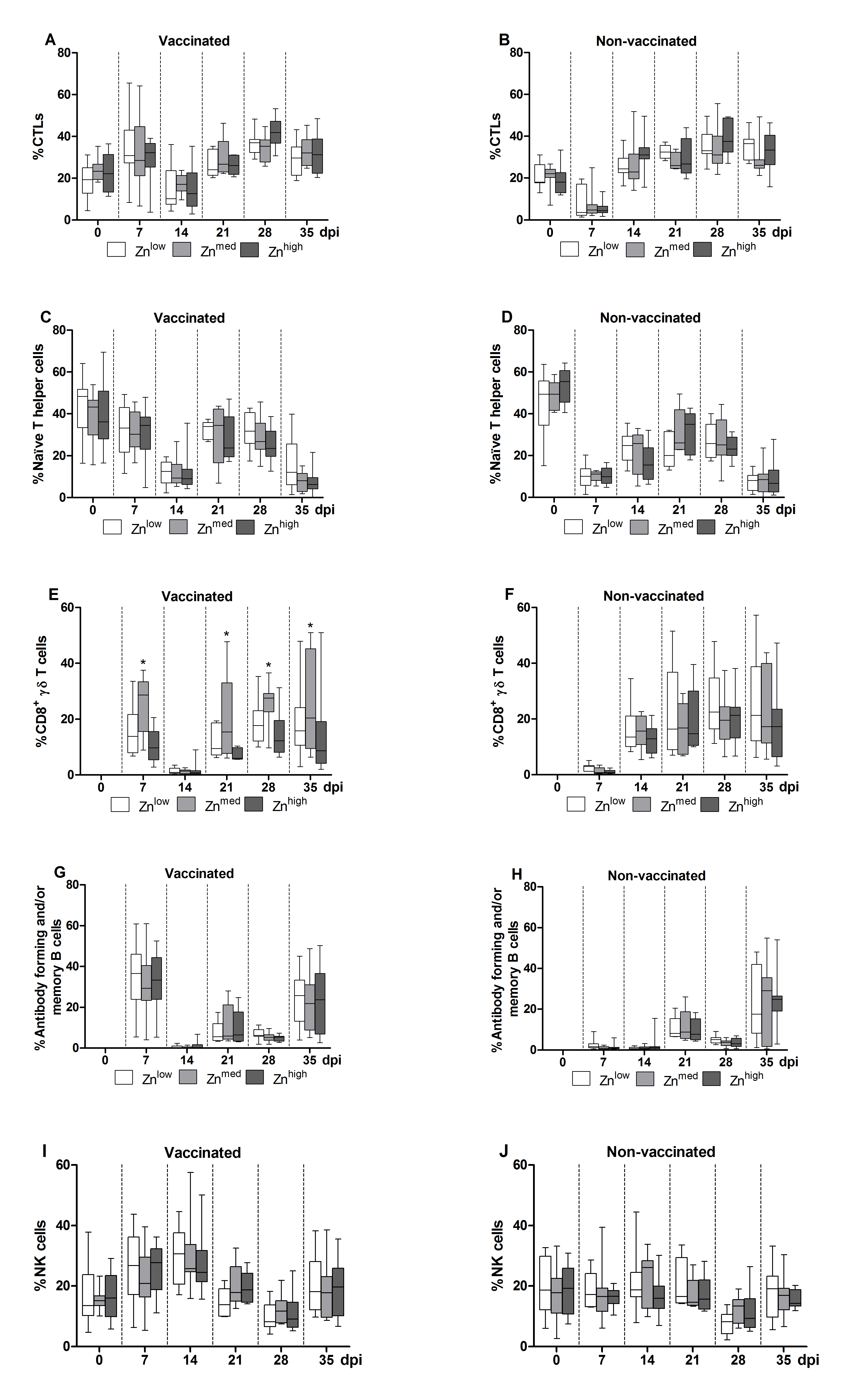

Supplement: Supplementary file 2 — Additional file 2: Figure S2: Modulation of PBMC immune cells frequencies determined by flow cytometry analysis. A and B, Cytotoxic lymphocytes (CD3+CD4−CD8 αhigh); C and D, naïve TH cells (CD3+CD4+CD8−); E and F, CD8+ γδ T cells (CD3+CD2+CD8+); G and H, Antibody forming and/or memory B cells (CD3−CD2+CD21−); I and J, NK cells. Asterisks indicate statistically significant differences (P < 0.05) between averages at each dpi. (JPEG 2 MB) [file 12985_2014_2469_MOESM2_ESM.jpeg]
